# Functioning of chronically ill patients receiving long-term care in Poland

**DOI:** 10.3389/fpubh.2025.1696868

**Published:** 2025-11-28

**Authors:** Bożena Majchrowicz, Krystyna Kowalczuk, Alicja Kłos, Katarzyna Tomaszewska

**Affiliations:** 1 Collegium Medicum, Institute of Nursing, University of Rzeszów, Rzeszów, Poland; 2 Department of Integrated Medical Care, Medical University of Białystok, Białystok, Poland; 3 Department of Nursing, Institute of Health Protection, The Bronisław Markiewicz Academy of Applied Sciences, Jarosław, Poland

**Keywords:** patient, home care, Barthel scale, functioning in chronic illness, depressive symptoms

## Abstract

**Introduction:**

Chronic diseases and disabilities require the implementation of multidimensional supportive measures – medical, social, and organizational. In Poland, home care plays a key role, enabling individuals with chronic illnesses to remain in their natural environment, which improves quality of life, independence, and social interaction.

**Aim:**

The aim of the study is to characterize the functioning of individuals with chronic diseases in the context of long-term care and to analyze selected determinants of this functioning. This assessment is important, as it enables the identification of factors that influence the quality of life and independence of patients receiving this form of care.

**Materials and methods:**

The study was conducted among patients receiving long-term home nursing care in south-eastern Poland. The diagnostic survey method was applied, using a questionnaire that included questions on sociodemographic data as well as standardized research tools: the Barthel Index, the Functioning in Chronic Illness Scale (FCIS), and the Beck Depression Inventory (BDI) - tools with established reliability and validity in chronically ill populations. For statistical analysis, the Mann–Whitney U test and Spearman’s rho correlation coefficient were used. Statistical significance was set at *p* ≤ 0.05.

**Results and conclusions:**

The mean score obtained during the last assessment was 19.38 (SD = 13.51), with a median of 15.00 points, indicating a low level of independence among the respondents. The overall functioning in chronic illness (FCIS) reached an average score of 59.02 (SD = 10.12). The mean severity of depressive symptoms in the study group was 19.2 ± 6.9 points on the Beck Depression Inventory, corresponding to moderate symptom severity. The study demonstrated significant associations between the level of patient independence, the occurrence of depressive symptoms, and functioning in the course of chronic disease. Most respondents presented a low level of independence, which translated into limited coping abilities, reduced functioning, and a considerable impact of illness on their daily lives and attitudes. Moreover, a substantial proportion of respondents exhibited moderate depressive symptoms, which may further worsen prognosis. The findings confirm the need for comprehensive, multidimensional care for patients with chronic diseases.

## 1 Introduction

The World Health Organization (WHO) defines chronic diseases as conditions that persist over a long period of time and require a systematic and long-term approach to treatment. These conditions generally do not fully respond to therapy and may limit daily activities. According to the WHO, chronic diseases include: non-communicable diseases (cardiovascular diseases, cancer, and diabetes); chronic infectious diseases (HIV/AIDS); certain mental disorders (depression and schizophrenia); and permanent structural impairments (amputations, blindness, and joint diseases) (1).

Chronic diseases pose a significant health and social challenge worldwide, as they are characterized by a prolonged course, limit daily functioning, and diminish quality of life. They require comprehensive care that includes pharmacological treatment as well as psychosocial support and rehabilitation. Long-term care, delivered in home or institutional settings, aims to maintain the highest possible level of functioning and independence for the patient while simultaneously supporting the family. In the context of population aging and the growing number of individuals with limited capacity for self-care, the importance of these services is steadily increasing (2–4).

Long-term care (LTC) refers to a set of medical and non-medical services designed for individuals of different ages whose ability to care for themselves is diminished due to aging, chronic conditions, or disability. These services may be delivered at home (home care), within community care, or in institutions such as nursing homes or hospices. Their overarching aim is to maintain the highest possible level of patient functioning while also supporting their families. With population aging, the demand for comprehensive LTC services is increasing—ranging from home and community-based care, through health monitoring, to rehabilitation and supportive therapies. A key value of these services is the preservation of older adults’ internal resources and functional capacity, with full respect for their rights, freedoms, and dignity. In light of ongoing transformations of healthcare systems—shifting from disease-oriented models to holistic approaches—the role of LTC in sustaining the quality of life of older adults is gaining significance. For this reason, the WHO emphasizes the necessity of health promotion, preventive measures, maintenance of functional abilities, and universal access to long-term care (5, 6).

The decline in the ability to perform activities of daily living, often referred to as “functional deterioration,” represents a significant health challenge in aging societies. Limited physical fitness is one of the main reasons for institutional admission. In the context of long-term care, the inability to perform basic daily activities has a strong impact on the demand for resources and the organization of services. Properly tailored interventions can slow down or even partially reverse this unfavorable trend. Even better outcomes can be achieved through precise forecasting of individual trajectories of functional decline (7–9).

Living with a chronic disease poses a considerable challenge for patients, as it disrupts various physical, psychological, and social functions, ultimately affecting their quality of life. Chronically ill patients face serious problems such as higher treatment costs, social isolation and loneliness, disability, fatigue, pain/discomfort, as well as feelings of anxiety, anger, hopelessness, frustration, fear, and depression (10). Individuals suffering from chronic illnesses experience major difficulties that impact both their physical and emotional health, and thus their daily functioning. It is well established that people with chronic diseases are more likely than the general population to experience mental health problems (11).

Disability and population aging increase the demand for healthcare, with expenditures being particularly high in the last year of life. Verified trends indicate the need to reconsider society’s response to the needs of this group. Healthcare systems must be rethought to strengthen and enhance their capacity to respond to diverse and growing health challenges, while ensuring appropriate, high-quality, and safe care tailored to the needs and preferences of patients (12). Understanding the relationships between the level of independence, emotional state, and functioning of patients receiving long-term care is crucial for planning effective caregiving and therapeutic interventions. This approach enables not only appropriate tailoring of the scope of care, but also early detection of psychosocial problems and the implementation of preventive measures.

The aim of the study was to assess the functioning of individuals with chronic diseases receiving home-based long-term nursing care and to analyze selected factors affecting their level of independence, the severity of depressive symptoms, and overall functioning in chronic illness. The study sought to determine the relationships between the physical and psychological aspects of patients’ functioning and to identify areas requiring particular support within long-term care. Based on the main objective, the following specific research questions were formulated:

1. Is there a relationship between the results of the self-care assessment scale and functioning in chronic illness?
2. Is there a relationship between the occurrence of depressive symptoms and the results of the FCIS scale?

## 2 Materials and methods

### 2.1 Research design

The study was conducted among patients receiving home-based long-term nursing care in the Subcarpathian Voivodeship in Poland. Services were provided by non-public healthcare facilities holding contracts with the National Health Fund for the provision of home-based long-term nursing care. The study was cross-sectional and carried out in the second half of 2024. A diagnostic survey method was applied using a questionnaire technique. The research instrument was a questionnaire comprising items on sociodemographic data and standardized assessment scales: the Barthel Index, the Functioning in Chronic Illness Scale (FCIS), and the Beck Depression Inventory (BDI).

### 2.2 Research tools

#### 2.2.1 Barthel index

The Barthel Index is used to assess the patient’s level of independence in performing basic activities of daily living. It was selected due to its wide application in evaluating the functioning of individuals with chronic conditions and its high validity and reliability in populations receiving long-term care. Based on the score (ranging from 0 to 100), the level of functioning and the need for care can be determined. The diagnostic value and applicability of the Barthel Index in the Polish healthcare system have been confirmed in scientific studies.

The tool demonstrates high reliability and reproducibility of results, as evidenced by a Cronbach’s alpha coefficient ranging from 0.78 to 0.89, and a test–retest correlation coefficient in the range of *R* = 0.93–0.95. In clinical and institutional practice (e.g., in the qualification process for long-term care), particular significance is attributed to scores within the 0–40 range, which indicate severe functional deficits and justify the need for continuous care (13).

#### 2.2.2 Functioning in chronic illness scale (FCIS)

The Functioning in Chronic Illness Scale (FCIS) is an originally Polish tool developed by Aldona Kubica, designed for a comprehensive assessment of the functioning of patients with chronic diseases. This instrument enables a comprehensive evaluation of physical, emotional, and social aspects of functioning, which aligns with the primary objective of the study. The scale has been validated for reliability and validity (Cronbach’s *α* = 0.855). The questionnaire consists of 24 items divided into three subscales:

- *Impact of the disease on the patient* – subjective assessment of limitations resulting from the illness.
- *Impact of the patient on the course of the disease* – beliefs regarding responsibility and agency.
- *Impact of the disease on the patient’s attitude* – outlook on the future and acceptance of the illness.

Responses are given on a five-point Likert scale (1 = “definitely not” to 5 = “definitely yes”). The maximum score for each subscale is 40 points, and 120 points for the entire scale. Interpretation is based on percentile ranges:

- 0–78 points – low functioning.
- 79–93 points – medium functioning.
- ≥94 points – high functioning.

Analogous cut-off values are applied to each subscale. The total score provides an overall measure of patient functioning with chronic illness: the higher the score, the better the functioning in the assessed domains. The FCIS may be used both in scientific research and in clinical practice, particularly in long-term care (14).

#### 2.2.3 Beck depression inventory (BDI)

The Beck Depression Inventory (BDI) is one of the most widely used self-assessment tools for depression worldwide. It is used to measure the severity of depressive symptoms, which constitute a significant factor affecting the functioning of individuals with chronic illnesses. It was developed by Aaron T. Beck et al. in 1961 and has since undergone multiple modifications and cultural adaptations, including in Poland. The Cronbach’s *α* coefficient of the Polish adaptation is 0.92 (15).

The BDI is based on the cognitive theory of depression, which emphasizes negative thoughts about oneself, the world, and the future. It is a self-report tool in which patients independently assess the severity of depressive symptoms by choosing responses that best describe their state over the past week.

The BDI encompasses three main domains:

- *Emotional* – sadness, low mood, guilt.
- *Cognitive* – low self-esteem, sense of failure, pessimism.
- *Somatic* – sleep difficulties, loss of appetite, fatigue.

The use of the above instruments enabled a comprehensive assessment of the patients’ functioning in the physical, psychological, and social domains.

### 2.3 Participants

The study included 234 patients receiving long-term home care. The inclusion criterion was informed consent to participate, the ability to establish verbal contact, and the capacity to perform a self-assessment of their condition, as well as being under home nursing care.

Paper-based questionnaires were prepared, and after obtaining consent from the care providers, the authors personally distributed them to institutions delivering home care services in Subcarpathian Voivodeship in Poland. During meetings with nursing staff, information was provided regarding the study’s purpose and instructions for completing the questionnaire, with a request to conduct the survey in patients’ home environments. A total of 350 questionnaires were distributed, of which 234 were correctly completed and returned after 2 months, yielding a response rate of 66.8%.

### 2.4 Ethical procedure

The study was conducted in accordance with ethical standards outlined in the Declaration of Helsinki (64th WMA General Assembly, Fortaleza, Brazil, October 2013) and Polish legal regulations. Approval was obtained from the Bioethics Committee of PANS in Przemyśl (approval number: KBPANS/15/2024).

### 2.5 Statistical analysis

Statistical analysis was performed using the Mann–Whitney U test and Spearman’s rho correlation coefficient. A significance level of *p* ≤ 0.05 was adopted.

## 3 Results

The study included 234 patients receiving long-term home nursing care. All participants met the eligibility criteria for services funded by the National Health Fund. Sociodemographic data are presented in Table 1.

**Table 1:** Characteristics of the study group.

Variable		Frequency (*N*)	Percentage (%)
Gender	Female	164	70.1
	Male	70	29.9
Age (years)	26–35	10	4.3
	36–45	4	1.7
	46–55	36	15.4
	56–65	26	11.1
	More than 65	158	67.5
Place of residence	City	122	52.1
	Village	112	47.9
Duration of care (years)	Less than 1	34	14.5
	1–5	146	62.4
	6–10	50	21.4
	More than 10	4	1.7
Who provides care in addition to the long-term home care nurse?	Immediate family (spouse, parents, siblings, children)	206	88.0
	Extended family	6	2.6
	Social welfare caregiver (MOPS/GOPS)	18	7.7
	The patient lives alone	4	1.7

The respondents indicated various medical conditions that had been diagnosed and constituted the basis for their inclusion in home-based long-term nursing care due to functional deficits. Among younger individuals (up to 55 years of age), the most common were injuries resulting from traffic accidents (8.5%), genetic disorders (1.8%), neuromuscular diseases (3.2%), and cancers (10.2%). Older individuals (over 55 years of age) were most often qualified for care due to a range of complications arising in the course of chronic diseases, including diabetes (8.5%), rheumatoid arthritis and degenerative diseases (14.5%). Dementia-related disorders were present in 12.8% of respondents. Additionally, many individuals had comorbidities that also contributed to reduced functional capacity.

### 3.1 Results of the Barthel index

Among the 234 participants, the majority were completely dependent (0–20 points on the independence assessment scale) – 150 individuals, representing 64.1% of the entire group. Partially independent individuals (21–40 points) accounted for 35.9%, corresponding to 84 participants. These data indicate that the vast majority of respondents required substantial assistance in daily functioning (Table 2).

**Table 2:** Scores obtained during the most recent assessment according to the Barthel Index.

Level of independence		Frequency (N)	Percentage (%)
Valid	Completely dependent (0–20 pts)	150	64.1
	Partially independent (21–40 pts)	84	35.9
	Total	234	100.0

### 3.2 Results of the functioning in chronic illness scale (FCIS)

Table 3 presents the results concerning four aspects of functioning among patients receiving long-term care, assessed using the FCIS and FCIC scales. In terms of overall functioning in chronic illness (FCIS), the vast majority of respondents (98.3%) obtained scores indicating a low level of functioning (≤78 points), reflecting significant difficulties in everyday functioning related to chronic disease. Only 1.7% achieved a medium level (79–93 points).

**Table 3:** Results of the FCIS scale.

Scale / domain	Level	Frequency (*N*)	Percentage (%)
FCIS – Functioning in chronic illness (Total)	Low level (≤78 pts)	230	98.3
	Medium level (79–93 pts)	4	1.7
	Total	234	100.0
FCIC – Impact of disease on the patient	Low level (≤23 pts)	222	94.9
	Medium level (24–33 pts)	12	5.1
	Total	234	100.0
FCIC – Impact of the patient on the disease	Low level (≤24 pts)	146	62.4
	Medium level (25–29 pts)	64	27.4
	High level (≥30 pts)	24	10.3
	Total	234	100.0
FCIC – Impact of disease on the patient’s attitudes	Low level (≤27 pts)	224	95.7
	Medium level (28–33 pts)	10	4.3
	Total	234	100.0

Analysis of the domain *impact of disease on the patient* (FCIC) showed that 94.9% of respondents obtained low scores (≤23 points), indicating a strong influence of the illness on various aspects of the patient’s life. Only 5.1% achieved a score in the medium range (24–33 points).

In the domain *impact of the patient on the disease* (FCIC), results were more diverse. A low level of engagement in coping with the disease was reported in 62.4% of respondents (≤24 points), a medium level in 27.4% (25–29 points), and a high level in 10.3% (≥30 points), suggesting limited resources or abilities to actively influence the course of illness.

With regard to the *impact of disease on the patient’s attitudes* (FCIC), low levels (≤27 points) predominated, with as many as 95.7% of respondents in this category. Only 4.3% achieved a medium level (28–33 points).

These findings indicate substantial limitations in the functioning of the studied population, as well as a strong, often negative impact of chronic illness on their daily lives, activity, and attitudes towards treatment and health.

### 3.3 Results of the Beck depression inventory (BDI)

The analysis showed that 16.2% of respondents did not present symptoms of depression (0–11 points on the BDI), while another 16.2% experienced mild depression. Symptoms indicative of mild severity were present in 16.2% of respondents; moderate symptoms were observed in 57.5%, while 10.3% exhibited very high symptom severity corresponding to severe depression on the Beck Scale (Table 4). These results confirm the considerable prevalence of depressive symptoms in the studied group of patients receiving long-term care.

**Table 4:** Results of the BDI scale.

Results	Frequency (*N*)	Percentage (%)
No depression (0–11 pts.)	38	16.2
Mild depression (12–19 pts.)	38	16.2
Moderate depression (20–25 pts.)	134	57.5
Severe depression (26 + pts.)	24	10.3
Total	234	100

The analysis demonstrated a moderate, statistically significant positive correlation: the impact of the disease on patient attitudes was significantly associated with the level of independence. This may suggest that patients’ attitudes towards their illness (e.g., acceptance, motivation) play an important role in their overall functioning. The strongest significant correlation (rho = 0.250**) was found between the impact of the disease on patient attitudes and independence. Other correlations were weak or statistically insignificant, suggesting that psychological attitudes may play a greater role in daily functioning than subjective perception of the illness itself (see Table 5).

**Table 5:** Correlations between Barthel Index scores and various aspects of functioning in chronic illness (FCIS).

Variable			Score obtained during the most recent assessment according to the Barthel Index
Spearman’s rho	FCIS – Functioning in chronic illness (TOTAL)	Correlation coefficient	0.151
	FCIC – Impact of disease on the patient	Correlation coefficient	−0.019
	FCIC – Impact of the patient on the disease	Correlation coefficient	0.100
	FCIC – Impact of disease on patient attitudes	Correlation coefficient	0.250^**^

**Correlation significant at the 0.01 level (two-tailed).

The Spearman’s rho correlation analysis revealed a moderate, statistically significant negative correlation. Better functioning in chronic illness was associated with lower levels of depression. The better patients managed daily life despite illness, the fewer depressive symptoms they experienced. The results suggest that subjective perceptions of illness and its impact on psychological wellbeing and attitudes are significantly related to the severity of depression. The strongest correlation was observed for the *impact of disease on the patient* (rho = −0.284). An active attitude toward the disease was not significantly correlated with depression level, which may indicate that patients’ behavioral actions (e.g., adherence to treatment) are not directly related to their emotional state (see Table 6).

**Table 6:** Spearman’s rho correlation analysis between BDI scores and selected aspects of functioning in chronic illness assessed with the FCIS.

Variable			Beck Depression Inventory (total score)
Spearman’s rho	FCIS – Functioning in chronic illness (TOTAL)	Correlation coefficient	−0.248^**^
	FCIC – Impact of disease on the patient	Correlation coefficient	−0.284^**^
	FCIC – Impact of the patient on the disease	Correlation coefficient	0.034
	FCIC – Impact of disease on patient attitudes	Correlation coefficient	−0.254^**^

**Correlation significant at the 0.01 level (two-tailed).

The analysis showed that age and duration of care were not significantly associated with the assessed variables. All rho values ranged between −0.165 and 0.079, which indicates very weak or no correlations. A weak correlation was found between age and the impact of disease on the patient (−0.165), which may suggest that older patients perceive the negative effects of illness more strongly; however, this relationship was not strong enough to allow generalizable conclusions (Table 7).

**Table 7:** Associations between age, duration of care, and various indicators of patient functioning, illness perception, and depression.

Variable			Age	Duration of care
Spearman’s rho	Barthel Index (last assessment score)	Correlation coefficient	−0.073	−0.047
	FCIS – Overall functioning in chronic illness	Correlation coefficient	−0.042	−0.058
	FCIC – Impact of disease on the patient	Correlation coefficient	−0.165	0.018
	FCIC – Impact of the patient on the disease	Correlation coefficient	−0.042	−0.032
	FCIC – Impact of disease on patient attitudes	Correlation coefficient	0.079	−0.100
	Beck Depression Inventory (total score)	Correlation coefficient	−0.033	−0.002

## 4 Discussion

The results of the study unequivocally confirm the existence of a relationship between the level of independence of patients receiving home-based long-term care and their functioning in chronic illness, as well as the severity of depressive symptoms. The findings are consistent with observations reported in the literature, which emphasize that limited independence is one of the main factors reducing quality of life and the capacity to adapt to chronic disease (16–18).

Similar conclusions are presented by researchers who underscore that the functioning of individuals with chronic illness deteriorates in the absence of social and psychological support (19). Additionally, according to studies conducted in a geriatric population by Starczewska et al. (20), low independence correlates with an increase in depressive symptoms and with the patient’s limited influence over the course of treatment.

The study demonstrated a significant association between patients’ level of independence and the severity of depressive symptoms—individuals more dependent on caregivers more frequently experienced low mood and feelings of helplessness. This result is consistent with observations by other authors, who stress that limited autonomy in daily functioning is a risk factor for depressive symptoms in people with chronic illnesses (21–23). At the same time, these studies indicate that emotional support, patient activation, and tailoring the scope of care to individual capabilities can mitigate the negative impact of dependence on mental health.

Our findings confirm the need for a holistic approach to patients receiving long-term care—integrating medical and psychological components to maintain independence and improve psychological wellbeing.

In the context of clinical practice, the results indicate the need to implement integrated care that accounts for both the physical and psychological dimensions of functioning among patients with chronic diseases. Support in the areas of psychological therapy, rehabilitation, and social activation may contribute to improved quality of life for these patients.

In the studies by Iovino et al., a lower level of self-care was associated with a higher risk of depressive disorders and poorer clinical prognosis, which is also reflected in our material. Similar conclusions are presented by Starczewska et al. and Beaudin et al., who indicate that deterioration in physical and emotional functioning among people with chronic diseases requires a comprehensive therapeutic approach encompassing both medical care and psychosocial support (21–23).

With regard to independence, the literature highlights the importance of cognitive and motivational functioning. Individuals who are more independent are inclined to engage in health-promoting behaviors, adhere to therapeutic recommendations, and undertake physical activity. Conversely, those who are entirely dependent more often experience feelings of helplessness and a lack of control over their lives, which may foster the occurrence of depressive symptoms (24). The study by Patel et al. (25) demonstrated a considerable impact of chronic diseases on activities of daily living and impairments in older adults, further confirming the public health significance of multimorbidity in the older adults. Therefore, there is an urgent need to ensure high-quality care for older people suffering from chronic illnesses.

In the present study, it was shown that, with increasing age among patients receiving home-based long-term care, the prevalence of chronic diseases and the severity of functional limitations rise. Older individuals more frequently experienced difficulties with self-care, which translated into a lower level of independence and poorer psychosocial functioning. This relationship confirms that aging in the context of chronic disease is a multidimensional process encompassing both physical and emotional aspects.

Convergent results were obtained by Iovino et al. (21) and Beaudin et al. (23), who emphasize that the accumulation of chronic diseases and the associated functional limitations lead to a decline in quality of life and an increased risk of depressive symptoms. Sharma et al. (26) further indicated that late-onset chronic diseases—especially stroke or cancer—are associated with heightened physical inactivity and poorer psychological well-being among older adults. These results align with the observations obtained in the present study and confirm that age, multimorbidity, and functional status are key determinants of the level of functioning among patients receiving long-term care.

Social support is also a crucial aspect of this issue. As other studies show, individuals with strong social support demonstrate better health outcomes and are less susceptible to depression (27). In the context of chronic illness, such support may take the form of assistance with daily activities, emotional support, or access to healthcare resources.

In this study, the mean severity of depressive symptoms was 19.2 ± 6.9 points on the Beck Depression Inventory, indicating a moderate level of depression among patients receiving home-based long-term nursing care. This finding confirms that limited independence and long-standing chronic illness significantly diminish the psychological wellbeing and functional status of the individuals studied. Similar observations were presented by Sharma et al., who, analyzing a population of older adults in India, demonstrated that both early and late onset of chronic diseases (single or multiple) were associated with deteriorating physical condition, functional limitations, and an increased risk of physical inactivity. The authors emphasized that, in particular, the late onset of conditions such as stroke or cancer had a stronger association with reduced activity and wellbeing, which may indicate the need for early identification of high-risk groups and the implementation of preventive measures (26).

The convergence of these observations with the results of the present study confirms that physical, emotional, and functional factors are interrelated elements of the chronic disease process, and their comprehensive assessment should form the basis for long-term care planning. Pawłucka found that mood disorders adversely affect both the course of chronic disease and the effectiveness of treatment, as well as patients’ quality of life and treatment costs. Moreover, the complex etiology of depression among patients with chronic diseases residing in institutional care facilities underscores the importance of a holistic approach to diagnosis and the need for an interdisciplinary approach to treatment (28). These differences may stem from variations in the care context (institutional vs. home-based), sample structure, and the measurement instruments used. Accounting for these factors allows for a more complete understanding of the complex nature of functioning among patients with chronic diseases and confirms the need for further research in this area.

The results obtained in the study population may point to significant gaps in the long-term care system, particularly in the areas of psychological and rehabilitative support. Addressing emotional needs and promoting patients’ independence should be key elements of therapeutic plans. The ability to influence the course of one’s own illness is not only beneficial from a psychological perspective but also contributes to better treatment outcomes.

It is recommended that the assessment of independence and depression levels be adopted as standard practice in the care of patients with chronic disease. This enables early detection of problems and the tailoring of care to the patient’s individual needs. The introduction of psychotherapeutic, educational, and community-based interventions can effectively reduce the severity of depressive symptoms and improve patients’ overall functioning. A review of the literature indicates that integrating medical, rehabilitative, and psychosocial services within long-term care significantly improves the quality of life of patients with chronic diseases. Early implementation of rehabilitative interventions and psychological support is essential, as these reduce symptoms of depression and anxiety. Telemedicine opens new possibilities for remote monitoring and support, although it requires appropriate infrastructure and digital competencies on the part of both patients and staff.

## 5 Conclusion

The study demonstrated significant associations between patient independence, depressive symptoms, and functioning in the course of chronic illness. The majority of respondents exhibited low levels of independence, which translated into limited ability to cope with illness, low functioning, and a considerable impact of disease on daily life and attitudes. Moreover, a substantial proportion of respondents displayed moderate to severe depressive symptoms, which may further worsen prognosis and quality of life. At the health policy level, it is recommended to further develop the model of home-based long-term nursing care by expanding access to services, standardizing functional assessment instruments, and training personnel to identify depressive symptoms. The findings confirm the need for comprehensive, multidimensional care for patients with chronic illnesses.

## 6 Limitations of the study

This study has several limitations that should be considered when interpreting the findings. Its cross-sectional design does not allow for the establishment of causal relationships between the analyzed variables. Furthermore, the sample was limited, as the study was conducted in only five long-term care facilities, which restricts the generalizability of the results to the broader population of chronically ill patients. Another limitation arises from the use of self-report measures, including the Beck Depression Inventory (BDI). Such tools, while widely recognized, are inherently subjective and may be influenced by external factors such as the respondent’s current mood or motivation.

## 7 Implications for practice

The results of this study have several important implications for nursing practice. A holistic approach to patient care is essential, requiring nurses to consider both physical and psychological aspects of health, with particular attention to depressive symptoms and the level of independence. Early identification of depression risk should be prioritized, and the regular use of screening tools such as the Beck Depression Inventory may allow timely detection of problems and referral to appropriate specialists. Promoting independence through health education, patient motivation, and adaptation of the living environment can improve self-sufficiency and enhance daily functioning. Equally important is close interdisciplinary collaboration, where nurses work alongside psychologists, occupational therapists, and physicians to develop individualized care plans. Finally, involving families and caregivers in the care process is vital, as their participation can improve the effectiveness of interventions while reducing the psychological burden experienced by patients.

## Data Availability

The original contributions presented in the study are included in the article/supplementary material, further inquiries can be directed to the corresponding author.
